# Operational research in the context of advancing towards tuberculosis elimination in the Americas

**DOI:** 10.26633/RPSP.2019.101

**Published:** 2019-12-20

**Authors:** Jarbas Barbosa da Silva, Marcos Espinal

**Affiliations:** Pan American Health Organization/World Health Organization Pan American Health Organization/World Health Organization Washington, DC United States of America Pan American Health Organization/World Health Organization Washington, DC, United States of America; Communicable Diseases and Health Analysis Pan American Health Organization/World Health Organization Washington, DC United States of America Communicable Diseases and Health Analysis Pan American Health Organization/World Health Organization Washington, DC, United States of America

Tuberculosis (TB) is a major source of ill health, one of the top 10 causes of death worldwide and the leading cause of death from a single infectious agent, ranking above HIV/AIDS. TB is preventable, treatable, and curable. Timely diagnosis and treatment can cure most people who develop TB and curtail onward transmission (1). Treatment requires multiple drugs and requires from 6 months to 2 years depending on the type of TB (drug susceptible or resistant). These long, drug-treatment regimens are challenging for both patients and health care systems.

The Sustainable Development Goals propose ending the TB epidemic by 2030 (2). The World Health Organization’s (WHO) End TB Strategy provides a framework for reaching this goal with a patient-centered approach and inter-programmatic and intersectoral interventions (3). These interventions involve affected communities and address the social determinants of TB, which have a greater impact on vulnerable populations. They are also aligned with the Universal Access to Health and Universal Health Coverage Strategy (4). The Declaration of the United Nations High-Level Meeting on TB held in September 2018 renewed the commitment of WHO Member States to strengthen their national TB efforts, fighting against this disease by leveraging the existing global frameworks (5, 6).

In the Region of the Americas, TB persists as a public health problem with an estimated 289 000 new and relapsed cases in 2018 and an incidence rate of 29 cases per 100 000 population. These rates are far greater than the 5.3 cases per 100 000 population targeted for 2030 (7). In 2018, just 81% of the TB cases were notified, leaving a gap of 55 000 cases not diagnosed. Additionally, 10% of the notified cases were coinfected with HIV and 11 000 cases were resistant to the key first-line anti-TB drugs.

Countries in the Region of the Americas have been working to implement the End TB Strategy and the Regional Action Plan for the Prevention and Control of Tuberculosis (8), along with their national strategic plans. Recently, significant progress has been made by increasing political commitment; improving diagnostic capacity and case management, including for TB/HIV coinfection and comorbidities; focusing on vulnerable populations; involving civil society; and increasing operational research (9).

Still, robust efforts are needed to sustain and improve upon the gains made to date. Persistent challenges must be tackled by accelerating the implementation and expansion of new rapid molecular tests; strengthening treatment of latent TB infection; implementing new and shorter treatment regimens; introducing innovative approaches for vulnerable populations and the social determinants of TB; and developing operational research to inform on better policies and interventions (7).

The third of the three pillars of the End TB Strategy refers to research and innovation (10). A Global TB Research Strategy, developed by WHO in consultation with Member States and other stakeholders, will be considered for adoption at the 2020 World Health Assembly (11). In this context, operational research has been recognized as a key instrument for developing major TB control strategies and vital to strengthening health programs. The importance of health system and policy research to TB control has been recently highlighted (12, 13).

With the aim of contributing evidence and options towards TB elimination in the Region the Americas, the *Pan American Journal of Public Health* is publishing this special supplement on “Operational research in the context of advancing towards TB elimination in the Americas.” The Communicable Diseases Research Program of the Communicable Diseases and Environmental Determinants of Health Department, Pan American Health Organization, led the effort to produce this thematic series. It includes studies carried out as part of the Structured Operational Research and Training Initiative (SORT IT), a global partnership led by the WHO Special Program for Research and Training in Tropical Diseases (TDR), which aims to train participants to conduct and publish operational research and influence policy and practice (14). The issue comprises 10 original peer-reviewed research articles that highlight strategies for addressing TB prevention, treatment, and care in three countries of the Region of the Americas: Ecuador, considering specific populations such as children, multidrug resistant patients, and prisoners; Paraguay, covering factors associated with failures to TB treatment, TB mortality, and TB and diabetes mellitus comorbidity; and Suriname, looking at determinants of sputum smear non-conversion and factors associated with TB/HIV mortality in co-infected persons.

The results of these studies will provide policy makers and managers with evidence for improving quality, effectiveness, and coverage of national TB programs.
